# Sex-difference in bone architecture and bone fragility in Vietnamese

**DOI:** 10.1038/s41598-018-26053-9

**Published:** 2018-05-16

**Authors:** Lan T. Ho-Pham, Thao P. Ho-Le, Linh D. Mai, Tam M. Do, Minh C. Doan, Tuan V. Nguyen

**Affiliations:** 1 Bone and Muscle Research Group & Faculty of Applied Sciences, Ton Duc Thang University, Ho Chi Minh, Vietnam; 2 University of Technology Sydney (UTS), Sydney, Australia; 3 Department of Rheumatology, People’s Hospital 115, Ho Chi Minh City, Vietnam; 4 Department of Epidemiology, Pham Ngoc Thach University of Medicine, Ho Chi Minh, Vietnam; 5 Bone Biology Division, Garvan Institute of Medical Research, Sydney, Australia; 6 St Vincent’s Clinical School, UNSW Australia, Sydney, Australia

## Abstract

This study sought to define the sex-difference in trabecular and cortical bone parameters in Vietnamese individuals. The study involved 1404 women and 864 men aged between 20 and 86 years who were recruited from Ho Chi Minh City, Vietnam. Trabecular and cortical volumetric BMD were measured at the proximal tibia and proximal radius at 4%, 38%, and 66% points, using a peripheral quantitative computed tomography XCT2000 (Stratec, Germany). Polar strength strain index was estimated from cortical bone parameters. Changes in bone parameters were assessed by the multiple linear regression model. Among individuals aged 20–39 years, women had significantly lower peak trabecular BMD at both the radius (40%) and tibia (16%) than men, but the age-related reduction in trabecular BMD were similar between two sexes. For cortical BMD, peak values in women and men were comparable, but the age-related diminution was greater in women than men. At any age, polar strength strain index in women was lower than men, and the difference was mainly attributable to cortical bone area and total bone mass. We conclude that in the elderly, sex-related difference in trabecular BMD is originated during growth, but sex-related difference in cortical BMD is determined by differential age-related bone loss.

## Introduction

At any given age, women have higher risk of fracture than men^1^. However, factors underlying this gender-related difference are not well documented^2^. It has been postulated that differences in bone strength parameters are responsible for the gender-related difference in bone fracture. Bone strength is determined by both bone mass, its biomaterial composition, and its structural properties^3,4^. Currently, the most popular and “gold standard” method for the assessment of bone mass is dual-energy X-ray absorptiometry (DXA). The DXA technology can measure bone mass per area of bone and the resultant measure is called areal “bone mineral density” (aBMD). Extensive data over the past three decades reveal a common age-related evolution of BMD: that aBMD increases rapidly during the adolescent ages, reaches its peak between the age of 20 and 30^5^, and then declines rapidly after the age of 50^6^. An important feature of aBMD is that it is strongly associated with fracture risk^7,8^. Indeed, in the elderly, each standard deviation lower in aBMD was associated with about 2-fold increase in the risk of fracture^7^. Using the aBMD – fracture relationship data, an operational threshold of aBMD was developed for the diagnosis of osteoporosis^9^.

Although aBMD measurement has served the management of osteoporosis in clinical setting very well, it suffers from some limitations. Bone is a three dimensional construct, and the correct representation of bone mass should be volumetric BMD (BMD). However, aBMD measured by DXA is a two dimensional measurement, and as such, it does not fully capture the concept of bone strength. Two bones may have identical aBMD, but different volumetric BMD, because DXA could not take into account the thickness of the bone^10^. Moreover, aBMD measured by DXA does not distinguish between cortical and trabecular bone, in which their relative composition contributes differently to bone strength. In the elderly, the loss of connectivity of the trabecular bone together with the thinning of cortical shell in the cortical bone together cause age-related bone loss and reduced bone strength. However, the measurement of aBMD could not estimate the relative trabecular and cortical bone loss.

Peripheral quantitative computed tomography (pQCT) is a relatively new technology that can overcome the limitations of the DXA technology. The low-radiation pQCT allows a non-invasive estimate of BMD for trabecular and cortical bone parameters, and estimates of bone strength^11^. Previous studies have shown that cortical and trabecular structure parameters were strongly correlated with bone strength, and could help predict the risk of fracture in postmenopausal women^12–15^. However, the age-related changes in BMD and bone strength have not been well documented for Asian populations. Therefore, the present study was designed to fill the gaps in knowledge. We specifically sought to define the age-related changes in BMD, bone structural parameters and bone strength in men and women of Vietnamese background.

## Results

The study included 1404 women and 864 men aged between 20 and 99 years who were randomly recruited from Ho Chi Minh City and surrounding districts. The average age for women and men was 46 yr (SD 15) and 43 yr (SD 15), respectively. The proportion of women and men aged 50 years and older was approximately 48% and 39%, respectively (Supplementary Table 1). About one-third of men and women had tertiary education or higher. Just over 70% of participants were married, and 22% were single. As expected, the prevalence of current smoking in men (37%) was greater than that in women (0.7%). Furthermore, men were more likely to self-report as regular alcohol drinkers compared to women (47% vs 3%). There were 191 individuals on calcium and vitamin D and 31 on bisphosphonates.

The average BMI for women and men was 22.5 kg/m^2^ (SD 3.2) and 23.3 kg/m^2^ (SD 3.2), respectively. Approximately 7.5% of women and men had BMI greater than 27.5 kg/m^2^, the level deemed to be “obese” in Asian populations^16^. While lean body mass in men was 41% higher than women, fat mass in men was 15% lower than women. The percent of body fat, as expected, was higher in women than men (41.5% vs 30.3%, P < 0.001). Approximately 64% of women and 55% of men had percent body fat greater than 40% and 30%^17^, respectively.

As reported elsewhere, the prevalence of diabetes (as assessed by HbA1c) in this population was ~11% in both men and women combined^18^. Among those aged 50 years and older, approximately 14% of women and 5% of men had osteoporosis (i.e., femoral neck aBMD T-scores ≤ −2.5). In general, the baseline distribution of characteristics of participants was typical of a urban population in Vietnam.

### Age-related changes in bone parameters

The relationship between age and almost all measurements of volumetric BMD followed a third degree of polynomial model (Supplementary Table 2 and Supplementary Fig. 2). According to this model, BMD was cross-sectionally elevated between the age of 20 and 39, stable between the age of 40 and 50, and then declined after the age of 50. However, the magnitudes of change were different between men and women. The model with sex and age, age squared, and age cubed accounted for between 30% and 40% of the variance in areal BMD, between 40–44% of the variance in radius and tibia trabecular BMD at the 4% site, but only 15–25% of the variance in radius and tibia cortical BMD at the 38% and 66% sites (data not shown).

### Estimates of peak bone mass

Table 1 shows the determinants of peak BMD and associated parameters (among those aged 20–39 years). Body weight was the most consistent and the strongest predictor of BMD, bone area, and cortical thickness. Stature was also a significant predictor of trabecular BMD (but not cortical BMD), however the magnitude of association was modest. It is interesting to note that stature was negatively associated with trabecular BMD at the radius and tibia. After adjusting for weight and height, men had greater BMD and trabecular bone area than women. For a given weight and stature, men also had greater cortical bone area and cortical thickness than women. Collectively, the three factors – sex, weight and height – accounted for between 12 and 55% of variance of bone parameters.

**Table 1:** Determinants of peak radius and tibia bone parameters (aged 20–39 yr): multiple linear regression analysis.

	Regression coefficient (standard error)			
	Sex (Male vs Female)	Height (per cm)	Weight (per kg)	R^2^
**Proximal radius**				
Total BMD	46.2 (5.8)*	−1.09 (0.39)*	0.45 (0.25)*	0.12
Trabecular BMD @4%	63.8 (4.4)*	−1.32 (0.30)*	0.71 (0.19)*	0.34
BSI @4%	1991 (111)*	−10.3 (7.3)	21.1 (4.6)*	0.51
Cortical BMD @38%	0.73 (0.45)	0.22 (0.33)	1.05 (0.21)*	0.06
Trabecular bone area @4%	26.7 (2.8)*	0.78 (0.15)*	0.19 (0.09)*	0.48
Cortical bone area @38%	15.7 (1.1)*	0.29 (0.07)*	0.11 (0.05)*	0.49
Cortical thickness @38%	0.29 (0.04)*	0.005 (0.002)	0.003 (0.001)*	0.18
Polar SSI @38%	88.8 (4.0)*	0.60 (0.27)*	1.26 (0.17)*	0.41
**Proximal tibia**				
Total BMD	32.6 (4.1)*	−1.67 (0.28)*	1.09 (0.17)*	0.18
Trabecular BMD @4%	32.2 (3.4)*	−1.24 (0.23)*	0.96 (0.15)*	0.28
BSI @4%	3497 (251)*	−18.1 (11.6)	90.5 (10.5)*	0.49
Cortical BMD @38%	−15.5 (2.9)*	−0.18 (0.19)	0.65 (0.12)*	0.25
Trabecular bone area @4%	59.8 (4.9)*	3.39 (0.33)*	1.24 (0.21)*	0.63
Cortical bone area @38%	40.7 (3.5)*	0.84 (0.24)*	1.35 (0.15)*	0.55
Cortical thickness @38%	0.54 (0.06)*	0.001 (0.004)	0.011 (0.002)*	0.28
Polar SSI @66%	407.1 (39.3)	16.3 (2.6)	8.8 (1.6)	0.50

Note: Values are regression estimates and standard error (in brackets). *Denotes statistically significant at P < 0.01.

**Figure 1:**
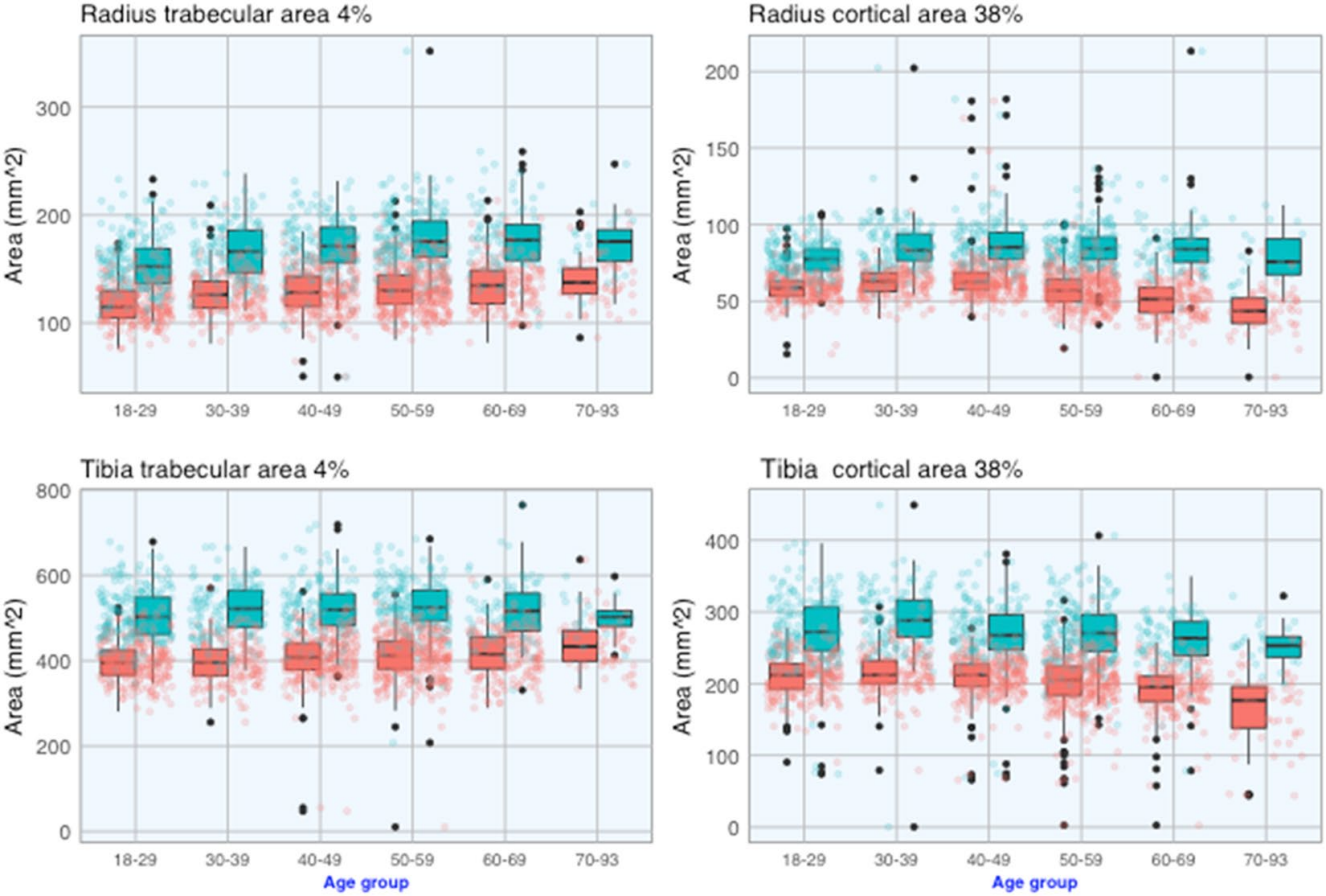
Age-related changes in trabecular (left) and cortical (right) bone areas at the proximal radius (top) and proximal tibia (bottom) in 1404 women (red) and 864 men (cyan).
- **Trabecular BMD**. Trabecular bone BMD was measured at the 4% site for the radius and tibia. The peak trabecular BMD (for individuals aged 20 and 39 yr) in women was ~15–20% lower than that in men (Table 2). However, the rate of lowering with age in BMD was comparable between men and women. For instance, between the age of 20–39 and 60–69, trabecular BMD in women was lowered by −23% at the radius and −15% at the tibia, and the corresponding reduction in men was −23% and −20% (Table 2 and Fig. 2).

To illustrate the sex-difference in the magnitudes of age-related changes, Table 2 shows the descriptive statistics for bone parameters among those aged between 20 and 39 years (i.e., peak values), and the percentage change in bone measurements between the age of 20–39 and 60–69. Some key observations from this analysis can be highlighted as follows:

- **Bone area**. At either the radius or tibia, the peak value of bone area in women was consistently and significantly lower than in men (Fig. 1), and the difference was about 25%. At the 4% and 38% radius sites, bone area tended to increase with advancing age, but the rate of increase was modest. For example, at the radius 4% site, bone area increased by ~6% between the age of 20–39 and 60–69 yr.

**Figure 2:**
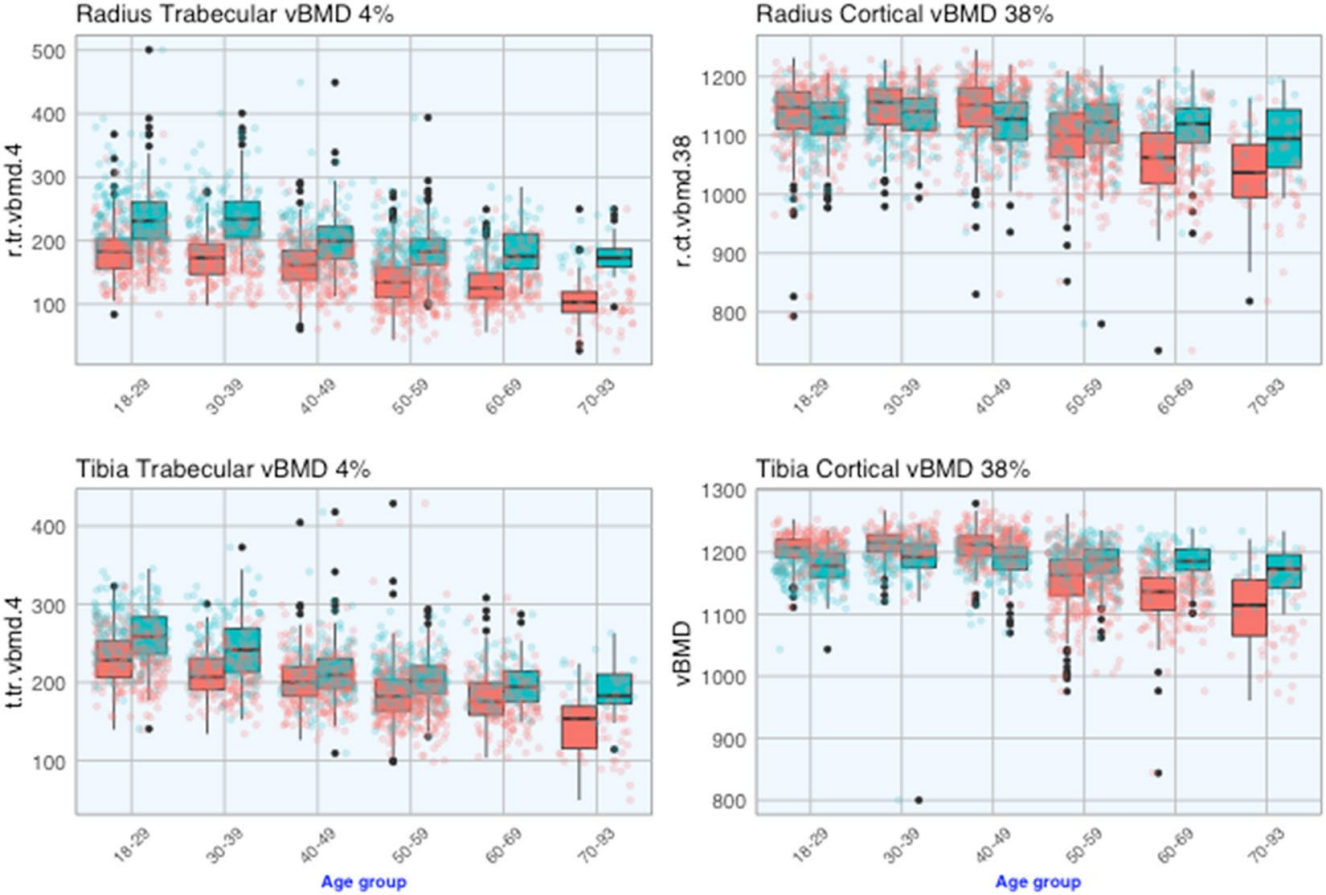
Age-related changes in trabecular (left) and cortical (right) BMD at the proximal radius (top) and proximal tibia (bottom) in 1404 women (red) and 864 men (cyan).
- **Cortical BMD**. Cortical bone BMD was assessed at the 38% site for the radius and 66% site for the radius and tibia. In contrast to the trabecular bone, there was no substantial difference in peak cortical BMD between sexes (Fig. 2). For instance, between the age of 20 and 39, BMD (4% portion of the radius) in women was 1146 ± 46 mg/mm^3^ which was slightly higher than that in men (1133 ± 41; *P* = 0.01). Similarly, tibia cortical BMD in women was also slightly higher than men at the 38% site (1214 ± 23 vs 1190 ± 42; *P* < 0.001) or 66% site (1173 ± 26 vs 1150 ± 27; *P* < 0.001) (Table 2).
  However, the age-related rate of lowering in cortical BMD in women was significantly higher than men (Fig. 2). Between the age of 20–39 and 60–69, cortical BMD [at either the radius 38% or tibia 38%] was lowered by approximately −7% in women, and this rate varied between −0.5 and −1.9% in men. As a result, after the age of 50, cortical BMD in women was significantly lower than men.
- The relationship between bone strength index (BSI) at the distal radius and distal tibia and age followed a 3rd order polynomial regression (Supplementary Table 2). Accordingly, BSI reached a plateau level between the age of 20 and 39, and gradually declined there after. For example, in women aged 60–69 yr, BSI at the distal radius was reduced by 34.9% compared with those aged 20–29 yr. The rate of reduction in radius BSI was comparable in men and women; however, at the distal tibia the rate of age-related reduction in BSI in women was greater than that in men (Table 2).

**Table 2:** Average percent of change in bone parameters between the age groups of 20–39 and 60–69.

Variable	Peak value between the age of 20–39: mean (SD)		Percent change between the age of 20–39 and 60–69	
	Women	Men	Women	Men
**pQCT radius @4%**				
Bone area	282.7 (42.5)	371.6 (58.2)*	6.0	6.5
Total BMD	351.9 (53.2)	406.5 (59.7)*	−24.1	−18.7*
Trabecular BMD	170.7 (35.9)	237.82 (47.4)*	−22.8	−23.1
BSI	8298 (1860)	12797 (3187)	−34.9	−32.4
**pQCT radius @38%**				
Bone area	120.6 (28.6)	162.9 (39.5)*	3.3	1.8*
Total BMD	757.0 (103.6)	734.2 (91.9)	−19.2	−2.5*
Cortical BMD	1146.0 (46.5)	1133.53 (41.3)	−7.4	−1.9*
Cortical thickness	1.96 (0.34)	2.27 (0.38)	−23.5	−1.3*
Fracture load	424 (126)	626 (137)	−7.3	−6.5
Polar SSI	194.8 (48.9)	297.8 (74.5)*	−5.6	−4.4*
**pQCT tibia @4%**				
Bone area	882.0 (106.8)	1152.4 (134.9)*	4.5	−0.7
Total BMD	300.4 (37.6)	329.8 (48.7)*	−19.3	−16.7
Trabecular BMD	210.9 (30.5)	243.8 (40.6)*	−15.2	−19.7
BSI	3531 (884)	5688 (1284)	−39.3	−24.8*
**pQCT tibia @38%**				
Bone area	328.0 (44.9)	428.5 (63.8)*	−0.9	−7.7*
BMD	872.6 (68.4)	873.7 (78.2)	−13.0	−2.6*
Cortical BMD	1213.8 (22.9)	1190.1 (41.6)	−7.0	−0.5*
Cortical thickness	4.18 (0.50)	4.87 (0.72)*	−11.1	−10.7
Polar SSI	1206.4 (223.4)	1742.2 (356.8)*	−12.0	−10.6
**pQCT tibia @66%**				
Bone area	458.8 (65.5)	600 (93.1)	−1.9	−7.0*
BMD	678.7 (73.6)	663.1 (74.4)	−13.2	−5.0*
Cortical BMD	1169.3 (26.0)	1145.1 (27.1)	−6.8	−0.8*
Cortical thickness	3.57 (0.45)	4.03 (0.56)	−14.6	−10.7
Fracture load	3100 (579)	4627 (938)	−13.6	−10.8
Polar SSI	1701.0 (322.1)	2407.7 (490.6)	−14.7	−10.3

BSI: Bone strength index; SSI: Strength stress index.

*Denotes statistically significant difference between men and women at P < 0.01.

#### Polar strength-strain index (SSI)

**Figure 3:**
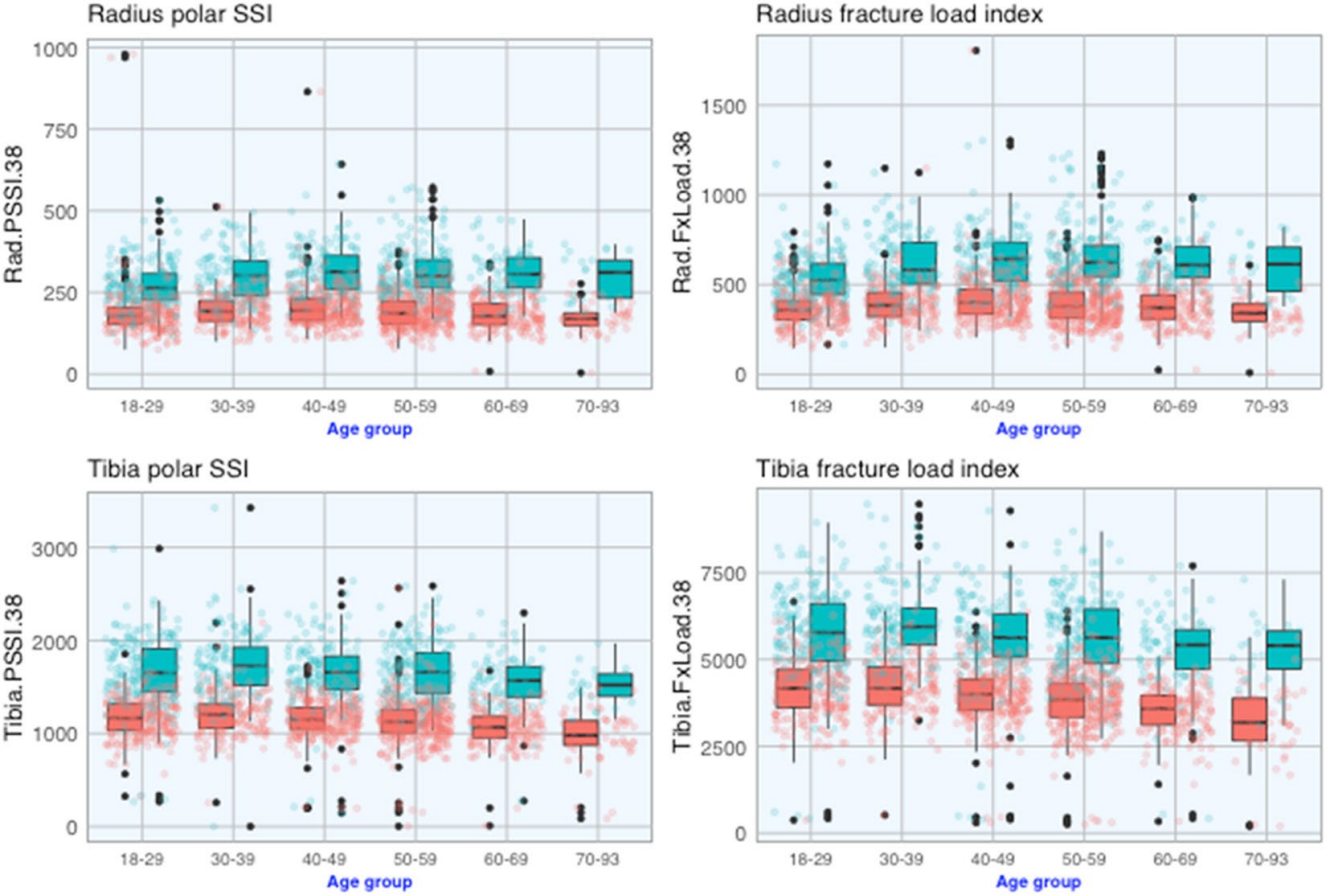
Age-related changes in polar SSI (left) and fracture load (right) at the proximal radius (top) and proximal tibia (bottom) in 1404 women (red) and 864 men (cyan).

Figure 3 shows that for any age group, polar SSI and fracture load index in men were significantly greater than those in women (at both radius and tibia 38%). There was a modest reduction in polar SSI with advancing age in both sexes.

Results of the Bayesian Model Averaging analysis of determinants of polar SSI are shown in Supplementary Table 3. The predictors of polar SSI identified by BMA were remarkably consistent for radius and tibia bones. Apart from age and sex, other pQCT measures were significant predictors: total bone area, total bone mass, total BMD, cortical bone area, cortical BMD, and cortical thickness (tibia 66% site). The factors accounted for ~81%, 45%, and 48% of total variance in polar SSI of radius 38%, tibia 38%, and tibia 66% site, respectively. In all indices of strength, bone area and cortical bone area and bone mass were important predictors, whereas total BMD or cortical BMD contributed a small proportion to the variation in bone strength indices.

## Discussion

It has been well established that women have a greater risk of fracture than men, but the reason for this difference has not always been clear. Guided by the hypothesis that fracture is directly related to bone strength which is, in turn, related to bone size and composition, the present study found that: (a) peak cortical BMD in men was the same as in women, but its age-related reduction was greater in women than men; (b) peak trabecular BMD in men was greater than women, but its age-related reduction was similar between men and women; and (c) bone size at the trabecular-rich bones tends to increase with advancing age, and the increase was greater in women than men, which leads to greater decrease in bone strength in women than men.

In this study, we observed that between the ages of 20 and 39, compared with women of the same age, weight and height, men had greater trabecular BMD and bone area at the radius and tibia. Men also had a greater bone sizes (as reflected by trabecular and cortical bone area) than women, even after adjusting for weight and height. These findings are in agreement with a previous study^19^ in which 18-year old men were associated with greater bone area and BMD than women of the same age, weight and height. Taken together, the differences in skeletal size and BMD between men and women are not entirely explained by body size, but perhaps by other factors, including hormones and genetic factors^3^.

We found that age-related reduction in BMD were substantially different between types of bone and sexes. At the proximal radius, trabecular BMD was higher in men than in women for every age group; in either gender, there was a relatively stable trabecular BMD until midlife and then lowered thereafter, but the rates of lowering were similar between genders, and these trends are consistent with previous studies using HRpQCT^20–22^. Between the ages of 30 and 90 years, the rate of lowering in trabecular BMD was ~23% for both sexes. Interestingly, between the ages of 20 and 60 years, cortical BMD in women and men was comparable, but after the age of 60 years, men had a greater BMD than women, because the rate of lowering in men was lower than women, and this finding is also in agreement with a previous study using HRpQCT^21^. Taken together, at the radius, the age-related reduction in cortical BMD was greater in women than men, but the age-related reduction in trabecular BMD was similar between men and women.

The patterns for the proximal tibia were not much different from those of the radius. Between the age groups of 30–39 and 60–69, cortical BMD in women was lowered greater than men, and this was mainly due to the decrease in cortical thickness in women was greater than men. In a previous study using micro-CT scan electron microscopy, trabecular BMD and BV/TV of the tibia declined with age more rapidly in women than men^23–25^. Thus, at the proximal tibia, the age-related decline in cortical BMD in women was greater than men, but the decline in trabecular BMD was not much different between women and men.

At both the proximal radius and proximal tibia, the estimated peak value of polar strength stress index in men was 40–50% higher than women. However, the age-related reduction in SSI was quite similar between sexes, and as a result, men had higher SSI than women for every age group. Moreover, BSI in men was greater than in women by approximately 50% to 60%, but the age-related rate of reduction in BSI was not much different between sexes. We also found that statistically, cortical bone area, total bone area and total bone mass were main determinants of SSI at the radius or tibia. However, the proportion of variance explained was greater for the proximal radius (81%) than for the proximal tibia (45%). This is an interesting finding because the formula for determining SSI does not involve total bone mass or cortical bone area. However, since SSI is highly correlated with fracture load, the sex-related difference in SSI implies that men have a lower risk of fracture than women because men have larger bone area and more bone mass than women.

The present study’s findings must, however, be considered within context of strengths and weaknesses. First, the study represents one of the largest studies of osteoporosis in Asian populations, and as such, it increased the reliability of estimates of peak bone mass and prevalence of osteoporosis. Second, the study population is highly homogeneous, which reduces the effects of potential confounders that could compromise the estimates. The criteria of inclusion were very broad which covers the wide range of subgroups in the general population. Nevertheless, the study also has a number of potential weaknesses. The participants in this study were sampled from an urban population; as a result, the study’s finding may not be generalizable to the rural population. Ideally, age-related changes in bone density should be estimated from a longitudinal study in which a large number of men and women is followed from the age of 5 till the age of 90, but such a study is not practically feasible. Therefore, estimates of aged-related changes in BMD in this study could overestimate the true changes^26^ and/or biased by cohort effects.

In summary, data from the Vietnam Osteoporosis Study suggest that bone fragility during senescence is determined mainly by age-related trabecular bone loss and reduced cortical bone mass. The sex-related difference in trabecular bone density is originated during growth (and may be before birth)^27^, but the sex-related difference in cortical bone density during old ages is resulted from age-related differences in bone loss.

## Study Design and Methods

The Vietnam Osteoporosis Study (VOS) was designed as a population-based family study. Participants were drawn from multiple families who were living in Ho Chi Minh City and surrounding rural areas. The study’s design rationale have been described elsewhere^28^. The procedures and protocol of VOS were approved by the research and ethics committee of the People’s Hospital 115. The Study was conducted according to the ethical principles of the Declaration of Helsinki, and all participants gave written informed consent.

The inclusion criteria were broad: men and women aged between 18 years and older, who agreed to participate in the Study. We excluded individuals deemed to have impaired cognitive function or are not willing to give informed consent or were physically unable to complete clinical tests. We used two approaches to recruit participants. In the first approach, we contacted community organizations to solicit a list of members, and from the list we ran a computer program to randomly selected individuals who met the age and gender criteria. A letter was then sent to the selected individuals to invite them and their family members to participate in the Study. In the second approach, we recruited participants via television, the Internet, and flyers in universities. The flyers (in Vietnamese) described the Study’s purposes, procedures, benefits and potential risks of participants. Individuals agreed to participate in the study were then transported to the Bone and Muscle Research Laboratory at the Ton Duc Thang University for clinical assessment and evaluation. Participants did not receive any financial incentive, but they received a free health check-up, and lipid analyses.

Each participant was administered with a structured questionnaire by a trained interviewer. The questionnaire solicits information concerning their demographic factors, socio-economic status, clinical history, medication use, lifestyle factors, physical activity, dietary habits, history of falls and fractures, and anthropometric factors. Height and weight were measured by an electronic portable, wall-mounted stadiometer (Seca Model 769; Seca Corp, CA, USA) without shoes or ornaments or hats or heavy layers of clothing. Body mass index (BMI) was derived as the weight in kilograms divided by the square of the height in meters. We classified the body mass index into 4 groups as follows: underweight (if BMI < 17); normal (BMI between 17 and 22); overweight (BMI between 23 and 27.5); and obese (BMI ≥ 27.5)^16^. Waist circumference (WC) and hip circumference (HC) were also measured in each participant by using the WHO protocol^29^. HC was measured around the widest portion of the buttocks (in standing position) by using a measuring tape. WC was measured at the midpoint between lower margin of the least palpable rib and the top of the iliac crest. Waist to hip ratio (WHR) was derived as the ratio of WC over HC. Central obesity was defined as WHR >0.85 for women or >0.90 for men.

### pQCT measurements

Measurements of trabecular and cortical bone parameters were done with a pQCT scan XCT-2000 (Stratec Medizinetechnik, Pforzheim, Germany) by a qualified radiographer. The scanning procedure strictly followed the manufacturer’s instructions, in which a planar scout scan was first determined to delineate the anatomic reference line for the non-dominant radius and tibia. If an individual has a prior fracture at the radius or tibia, the scan was done at the non-fracture site. The forearm length was determined from the elbow to the ulna styloid process, and the tibia length was determined from the proximal aspect of the medial condyle to most distal aspect of the medial malleolus of the tibia. A single slice of 2.5 mm thickness with a voxel size of 0.5 mm and a speed of 20 mm/s was taken at all locations. The scan image was processed by the radiographer using the Stratec software version 6.2. Daily phantom scans were analyzed to ensure long-term reproducibility. Two slices were taken in the radius at the 4% and 38% sites, and three slices were taken in the tibia at the 4%, 38%, and 66% sites. The 4% of the radius and tibia represent mainly trabecular bone, whereas the 38% and 66% portions represent mainly cortical bone. At the 4% site of the radius and tibia, we obtained total bone area (mm^2^), total and trabecular volumetric BMD (mg/cm^3^). At the 38% site, we obtained total bone area, total and cortical volumetric BMD, and cortical thickness (mm). In addition, at the 66% site, polar strain stress index (pSSI) with respect to the Y-axis (SSI; Supplement Fig. 1) was determined as follows^30^:

$$SSI=\sum _{i=0}^{n}({d}_{i}^{2}\times A\times (vBMD/vBM{D}_{max}))/{d}_{max}$$

where *d*_i_ is the distance from a cortical voxel to the x-axis; *d*_max_ is the maximum distance from the center of gravity to the outer voxel; *A* is the area of the voxel; *vBMD* is the density of the voxel; *vBMD*_max_ is the estimated physiological maximal cortical bone density (1200 mg/cm^3^). Previous studies found that SSI is a better index of bone fragility than moment of inertia^30,31^. Fracture load at the distal radius and distal tibia was estimated by the method described by^32^ and was provided by the XCT-2000 system. In addition, we estimated the bone strength index (BSI) as the product of volumetric bone density and bone area^33^ for the distal radius and tibia at the 4% portion.

### Data analysis

We used the simple linear regression model to estimate the age-related changes in bone related traits (e.g., BMD, bone strength indices). In this model, each trait was modeled as polynomial function of age. Based on the R-squared value and t-test of parameter estimates, we decided the most “optimal” model for describing the relationship between bone traits and age. The usual assumptions of the model (i.e., normal distribution, homogeneity of variance, and independence) were inspected by a scatterplot of residuals against predicted values. We found that the distribution of all bone parameters approximated the normal distribution.

We used the multiple linear regression model to search for factors that were associated with polar strain stress index. Since there were many potential “candidate” predictors of each bone strength index, the number of potential models could be very large. We used the Bayesian Model Averaging (BMA) approach^34^ to determine factors that were associated with bone strength. This approach has been shown to have better performance compared to “traditional” approaches such as stepwise regression^35,36^. In the BMA approach, the linear regression model was fitted for 2^M^ (where M is the number of potential variables) competing models. The BMA averaged point estimates for regression coefficient over the all possible models. BMA produces a posterior probability of each possible model and posterior probability for regression coefficient associated with each predictor. The posterior probability is a function of a prior probability and the likelihood of a model. In this study, given the large number of predictors and there is little information available for eliciting prior distributions, we used the “uninformative” prior distributions, that a priori, make all models and parameters equally likely are appealing. All analyses were done with the R statistical environment^37^ and the BMA package^38^. The relative importance of each and combined predictors was assessed by the “lmg” method^39^ which decomposes the overall R-square value into individual effect.

### Data availability

The datasets generated during and/or analysed during the current study are available from the corresponding author on reasonable request.

## Electronic supplementary material

Supplementary Information
