# Supplementary material for: Sex-difference in bone architecture and bone fragility in Vietnamese
Source: Sci Rep. 2018 May 16;8:7707. doi: 10.1038/s41598-018-26053-9 (PMC5955960; doi:10.1038/s41598-018-26053-9)
Supplement: Supplementary file 1 — Supplementary Information [file 41598_2018_26053_MOESM1_ESM.docx]

**Supplementary Data**

**Sex-difference in bone architecture and bone fragility in Vietnamese**

Lan T. Ho-Pham*^1^, Thao P. Ho-Le^2^, Linh D. Mai^3^, Tam M. Do^4^,

Minh C. Doan^1^, Tuan V. Nguyen^1,2,5,6^

^1^Bone and Muscle Research Group & Faculty of Applied Sciences, Ton Duc Thang University, Vietnam;

^2^University of Technology Sydney (UTS), Sydney, Australia;

^3^Department of Rheumatology, People's Hospital 115, Ho Chi Minh City, Vietnam;

^4^Department of Epidemiology, Pham Ngoc Thach University of Medicine, Vietnam;

^5^Bone Biology Division, Garvan Institute of Medical Research, Sydney, Australia;

^6^St Vincent's Clinical School, UNSW Australia, Sydney, Australia.

**SUPPLEMENTS**

**Supplement Table 1. Baseline characteristics of 1404 women and 864 men in the study**

| **Variable** | **Women** | **Men** |
| --- | --- | --- |
| N | 1404 | 864 |
| Age group (n; %) |  |  |
| 18 to 29 yr | 258 (18.4) | 219 (25.3) |
| 30 to 39 yr | 190 (13.5) | 144 (16.7) |
| 40 to 49 yr | 305 (21.7) | 167 (19.3) |
| 50 to 59 yr | 394 (28.1) | 214 (24.8) |
| 60 to 69 yr | 192 (13.7) | 94 (10.9) |
| 70 yr and above | 65 (4.6) | 26 (3.0) |
| Height (cm)^1^ | 153.7 (5.5) | 164.9 (6.0) |
| Weight (kg) | 53.1 (8.0) | 63.3 (10.0) |
| Waist to hip ratio | 0.85 (0.08) | 0.92 (0.79) |
| Body mass index (kg/m^2^) ^1^ | 22.5 (3.2) | 23.3 (3.2) |
| Underweight (n; %)^2^ | 108 (7.7) | 42 (4.9) |
| Normal weight (n; %)^2^ | 1028 (73.2) | 601 (69.6) |
| Overweight (n; %)^2^ | 236 (16.8) | 200 (23.2) |
| Obese (n; %)^2^ | 32 (2.3) | 21 (2.3) |
| Current smoking (n; %) | 10 (0.7) | 323 (37.4) |
| Alcohol use (n; %) | 41 (2.9) | 403 (46.6) |
| METs hr/week (total)^3^ | 235.2 (196.4) | 265.7 (189.1) |
| METs occupational activities^3^ | 201.1 (145.2) | 245.2 (175.8) |
| METs home activities^3^ | 27.3 (48.0) | 12.9 (27.8) |
| METs recreational activities^3^ | 2.5 (13.2) | 3.8 (16.2) |
| METs transport^3^ | 2.2 (12.1) | 3.5 (10.3) |
| Falls (n; %) | 192 (13.7) | 99 (11.5) |
| History of fractures (n; %) | 210 (15) | 181 (21) |
| **Osteoporosis medication use** |  |  |
| Calcium and vitamin D (n; %) | 160 (11.4) | 31 (3.6) |
| Bisphosphonates (n; %) | 26 (1.9) | 5 (0.6) |
| **Educational attainment (n; %)** |  |  |
| Primary | 282 (20.1) | 108 (12.5) |
| Secondary | 706 (50.2) | 467 (54.0) |
| College and university | 416 (29.6) | 289 (33.5) |
| **Marital status (n; %)** |  |  |
| Single | 309 (22.0) | 226 (26.2) |
| Married | 1001 (71.3) | 625 (72.3) |
| Divorced | 27 (1.9) | 8 (0.9) |
| Widowed | 67 (4.8) | 5 (0.6) |

^1^Values are mean and standard deviation (in brackets). ^2^Weight categories were classified as follows: "underweight" if BMI <18.5; "normal" if BMI ≥18.5 and <25; "overweight" if BMI ≥ 25 and 30; "obese" if BMI ≥30. ^3^MET: metabolic equivalent.

**Supplements Table 2.** Relationship between bone mineral density and age: results of the multiple linear regression.

**Women**

| **Variable** | **Regression coefficient (standard error)** | | | **R-square** |
| --- | --- | --- | --- | --- |
|  | **Age** | **Age squared** | **Age cubic** |  |
| **Radius @4%** |  |  |  |  |
| Bone area | 5.29 (2.01) | -0.09 (0.04) | 0.0006 (0.0002) | 0.078 |
| Bone mass | 0.059 (0.006) | -0.001 (0.0001) | 0.000007 (0.00000009) | 0.141 |
| vBMD | 14.1 (2.42) | -0.33 (0.05) | 0.002 (0.0003) | 0.365 |
| Trabecular vBMD | 3.88 (1.66) | -0.11 (0.03) | 0.0007 (0.0002) | 0.253 |
| BSI | 321.9 (37.0) | -7.18 (0.80) | 0.044 (0.0054) | 0.352 |
| **Radius @38%** |  |  |  | 0.000 |
| Bone area | 0.035 (0.0085) |  |  |  |
| Bone mass | 0.005 (0.008) | -0.0000009 (0.0002) | 0.0000000005 (0.000001) | 0.11 |
| vBMD | 27.4 (4.72) | -0.56 (0.10) | 0.003 (0.0006) | 0.248 |
| Cortical vBMD | 20.0 (2.48) | -0.42 (0.005) | 0.002 (0.000004) | 0.272 |
| Cortical thickness | 0.10 (0.02) | -0.002 (0.0003) | 0.00001 (0.000002) | 0.217 |
| Fracture load | 15.9 (5.47) | 0.30 (0.11) | 0.0016 (0.0008) | 0.028 |
| Polar SSI | 8.01 (2.69) | -0.15 (0.06) | 0.0008 (0.0003) | 0.019 |
| **Tibia @4%** |  |  |  |  |
| Bone area | -1.07 (1.18) | 0.026 (0.012) | 0.00005 (0.0007) | 0.029 |
| Bone mass | 0.046 (0.016) | -0.001 (0.0003) | 0.000007 (0.000002) | 0.361 |
| vBMD | 5.97 (1.70) | -0.15 (0.03) | 0.0008 (0.0002) | 0.382 |
| Trabecular vBMD | -1.41 (0.06) | 0.026 (0.03) | -0.0002 (0.0002) | 0.282 |
| BSI | 263.4 (75.9) | -6.89 (1.65) | 0.041 (0.011) | 0.353 |
| **Tibia @38%** |  |  |  |  |
| Bone area | 0.008 (0.09) | 0.03 (0.04) | -0.002 (0.003) | 0.0006 |
| Bone mass | 0.066 (0.017) | -0.001 (0.0003) | 0.000007 (0.000002) | 0.153 |
| vBMD | 24.7 (4.13) | -0.50 (0.09) | 0.003 (0.0006) | 0.237 |
| Cortical vBMD | 21.5 (1.62) | -0.47 (0.03) | 0.003 (0.0002) | 0.488 |
| Cortical thickness | 0.044 (0.006) | -0.0006 (0.00007) | 0.000005 (0.000004) | 0.154 |
| Polar SSI | 5.78 (2.28) | -0.10 (0.02) | 0.002 (0.001) | 0.055 |
| **Tibia @66%** |  |  |  |  |
| Bone area | -0.15 (0.13) | 0.01 (0.007) | -0.00005 (0.0004) | 0.0001 |
| Bone mass | 0.058 (0.018) | -0.001 (0.0004) | 0.000007 (0.000002) | 0.228 |
| vBMD | 15.2 (3.88) | -0.32 (0.084) | 0.002 (0.0005) | 0.201 |
| Cortical vBMD | 18.2 (1.53) | -0.40 (0.03) | 0.002 (0.0002) | 0.496 |
| Cortical thickness | 0.03 (0.005) | -0.0005 (0.00005) | 0.000004 (0.000003) | 0.214 |
| Fracture load | 71.6 (26.7) | -1.48 (0.57) | 0.0079 (0.0039) | 0.080 |
| Polar SSI | 31.7 (14.2) | -0.13 (0.04) | 0.004 (0.002) | 0.087 |

**Notes:** values are estimated gradients and their standard error (in brackets)

**Men**

| **Variable** | **Regression coefficient** | | | **R-square** |
| --- | --- | --- | --- | --- |
|  | **Age** | **Age squared** | **Age cubic** |  |
| **Radius @4%** |  |  |  |  |
| Bone area | 4.77 (0.78) | -0.08 (0.066) | 0.0002 (0.0004) | 0.110 |
| Bone mass | 0.070 (0.01) | 0.001 (0.00001) | 0.000007 (0.000001) | 0.134 |
| vBMD | 12.5 (2.86) | -0.29 (0.06) | 0.002 (0.0004) | 0.226 |
| Trabecular vBMD | 7.40 (2.31) | -0.21 (0.05) | 0.001 (0.0003) | 0.232 |
| BSI | 464.7 (65.9) | -9.78 (1.49) | 0.058 (0.009) | 0.202 |
| **Radius @38%** |  |  |  |  |
| Bone area | 0.342 (0.092) | -0.05 (00.04) | 0.0002002 (0.00033) | 0.016 |
| Bone mass | 0.034 (0.012) | -0.0005 (0.0002) | 0.000002 (0.0000017) | 0.057 |
| vBMD | 2.57 (1.23) | -0.03 (0.01) | 0.0006 (0.0007) | 0.011 |
| Cortical vBMD | 6.51 (2.47) | -0.13 (0.05) | -0.0007 (0.0003) | 0.037 |
| Cortical thickness | 0.024 (0.005) | -0.0002 (0.00005) | 0.0004 (0.0003) | 0.025 |
| Fracture load | 15.12 (7.32) | -0.225 (0.161) | 0.0009 (0.001) | 0.043 |
| Polar SSI | 5.98 (0.96) | -0.06 (0.01) | 0.0009(0.0008) | 0.066 |
| **Tibia @4%** |  |  |  |  |
| Bone area | 5.47 (1.83) | -0.055 (0.02) | -0.0001 (0.0001) | 0.013 |
| Bone mass | 0.04 (0.03) | -0.001 (0.0006) | 0.000008 (0.000004) | 0.215 |
| vBMD | 3.96 (2.20) | -0.14 (0.05) | 0.001 (0.0003) | 0.287 |
| Trabecular vBMD | -1.65 (0.08) | -0.05 (0.04) | 0.0005 (0.0002) | 0.343 |
| BSI | 30.8 (15.0) | -9.82 (3.30) | 0.070 (0.022) | 0.277 |
| **Tibia @38%** |  |  |  |  |
| Bone area | -0.52 (0.14) | -0.15 (0.07) | 0.0009 (0.0005) | 0.016 |
| Bone mass | -0.11 (0.02) | -0.002 (0.0006) | 0.00001 (0.000004) | 0.047 |
| vBMD | 12.2 (4.57) | -0.24 (0.10) | 0.001 (0.0007) | 0.014 |
| Cortical vBMD | 5.06 (1.70) | -0.09 (0.04) | 0.0005 (0.0002) | 0.031 |
| Cortical thickness | 0.20 (0.009) | -0.0002 (0.0001) | 0.00001 (0.000005) | 0.014 |
| Polar SSI | 9.50 (4.35) | -0.13 (0.05) | 0.004 (0.002) | 0.021 |
| **Tibia @66%** |  |  |  |  |
| Bone area | 0.125 (4.9) | -0.25 (0.11) | 0.002 (0.0007) | 0.007 |
| Bone mass | 0.11 (0.03) | -0.0024 (0.0006) | 0.00001 (0.000004) | 0.085 |
| vBMD | -1.10 (0.19) | -0.08 (0.09) | 0.0006 (0.0006) | 0.041 |
| Cortical vBMD | 4.14 (1.54) | -0.083 (0.003) | 0.0005 (0.0002) | 0.030 |
| Cortical thickness | -0.009 (0.001) | -0.001 (0.0006) | 0.000009 (0.000004) | 0.055 |
| Fracture load | 98.7 (50.0) | -1.98 (1.10) | 0.011 (0.007) | 0.032 |
| Polar SSI | 11.76 (6.35) | -0.18 (0.07) | 0.009 (0.004) | 0.021 |

**Notes:** values are estimated gradients and their standard error (in brackets)

**Supplement Tabel 3. Contribution of bone parameters to polar strength strain index (SSI) of the radius and tibia at 38% and 66% sites**

| **Predictor** | **Radius polar SSI 38%** | **Tibia polar SSI 38%** | **Tibia polar SSI 66%** |
| --- | --- | --- | --- |
| Age | 0.4 | 0.9 | 1.6 |
| Sex | 10.4 | 16.8 | 14.6 |
| Bone area | 20.8 | 7.6 | 8.9 |
| Bone mass | 20.7 | 9.2 | 8.2 |
| Total vBMD | 2.3 | 1.2 | 2.2 |
| Cortical bone area | 24.2 | 8.5 | 7.9 |
| Cortical vBMD | 1.8 | 0.7 | 0.5 |
| Cortical thickness | 0.0 | 0.0 | 3.8 |
| Total R-squared | 80.6 | 44.9 | 47.8 |

Note: values represent R-squared value (in percentage) attributable to each predictor.

| **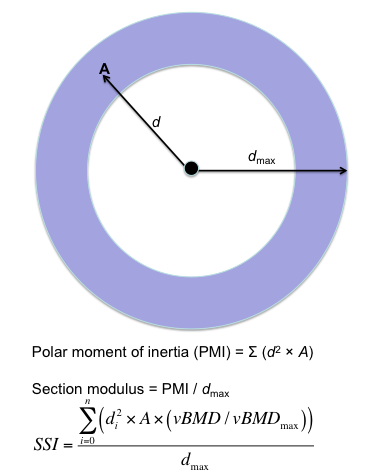** |
| --- |
| **Supplement Figure 1:** Definition of strength stress index (SSI) which is a function of the following measurements: the distance from a cortical voxel to the x-axis (*d*); the maximum distance from the center of gravity to the outer voxel (*d*_max_); the area of the voxel (A); density of the voxel (*vBMD*); and estimated physiological maximal cortical bone density (*vBMD*_max_ ; 1200 mg/cm^3^). |
